# Quality of life trajectories following geriatric hip fracture surgery: a growth mixture modelling approach

**DOI:** 10.1186/s13018-026-06664-z

**Published:** 2026-01-17

**Authors:** Tu Thai Bao Nguyen, Quang Son Tran, Thanh Tan Nguyen, Lien-Chen Wu, Yi-Jie Kuo, Yu-Pin Chen

**Affiliations:** 1 Department of Orthopedics, Faculty of Medicine, Can Tho University of Medicine and Pharmacy, Can Tho, 900000 Vietnam; 2 The International Ph.D. Program in Medicine, College of Medicine, Taipei Medical University, Taipei, 110 Taiwan; 3 Department of Orthopedics, Wan Fang Hospital, Taipei Medical University, No. 111, Sec. 3, Xinglong Rd., Wenshan Dist., Taipei, 116 Taiwan; 4 Department of Orthopedics, School of Medicine, College of Medicine, Taipei Medical University, Taipei, 110 Taiwan; 5 Graduate Institute of Biomedical Materials and Tissue Engineering, College of Biomedical Engineering, Taipei Medical University, Taipei, 100 Taiwan

**Keywords:** Hip fracture, Quality of life, Growth mixture modelling, Activities of daily living, Longitudinal study

## Abstract

**Background:**

Hip fractures remain a serious threat, particularly among the elderly. Characterizing longitudinal patterns of quality of life (QoL) is important to understand recovery after hip fracture. Identifying subgroups with similar trajectories can help design tailored rehabilitation strategies. This study aimed to investigate the trajectories of QoL over one year following hip fracture surgery and to identify factors associated with each trajectory class.

**Methods:**

This longitudinal study utilized a hip fracture registry database from a single medical center. QoL was assessed using the EuroQoL 5-dimension 3-level (EQ-5D-3L) questionnaire at admission, 6 months, and 1 year following hip fracture surgery. Growth mixture modelling was applied to identify subgroups of patients experiencing different trajectories of QoL. Baseline characteristics were compared between groups, and multivariate multinomial logistic regression was used to examine factors associated with trajectory group membership.

**Results:**

Three hundred and eighty-two patients with hip fracture (mean age 80.2; 71.3% female) experienced four distinct QoL trajectories after surgery that were identified: the *consistently high QoL* group (60.47%) maintained near-optimal QoL throughout follow-up, the *partially declined then stable* group (21.99%) showed a moderate drop at 6 months with stabilization by 1 year, the *notably decreased QoL* group (12.83%) demonstrated a steep and persistent decline, representing the poorest recovery, and the *recovered Qol* group (4.71%) started with the lowest baseline QoL but showed continuous improvement over one year. Multivariate multinomial regression showed that osteosarcopenia, a lower pre-fracture EQ-5D-3L utility score, and poorer activity of daily living predicted a *notably decreased QoL* trajectory, while older age and worse cognition predicted a *partially declined then stable* trajectory. No baseline covariates significantly predicted the recovered trajectory.

**Conclusion:**

Four QoL trajectories within one year following hip fracture surgery in elderly patients were explored. Age, comorbidity burden, musculoskeletal health, cognitive status, baseline QoL, and functional independence were significantly associated with trajectory membership. Routine assessment of these factors can help guide personalized management to help maintain QoL and prevent further decline.

## Introduction

Hip fracture remains a serious injury, often resulting in significant disability, morbidity, and mortality [1–3]. As the global population continues to age, the incidence of hip fractures is projected to rise substantially, placing a mounting burden on patients, caregivers, and healthcare systems [4–6]. Although advances in multidisciplinary perioperative care, surgical techniques, and rehabilitation programs have contributed to improved short-term survival, the long-term functional and psychosocial recovery remains highly variable and insufficiently addressed in many care pathways [7–9]. 

Quality of life (QoL) has emerged as a crucial outcome in geriatric care, reflecting a patient’s physical function, emotional well-being, social engagement, and autonomy [10–12]. Numerous factors have been identified as contributors to poor QoL, including the presence of multiple comorbidities, cognitive impairment or dementia, pre-existing functional limitations, musculoskeletal conditions such as osteoporosis and sarcopenia, and inadequate nutritional status [13–16]. These interrelated conditions can lead to a cascade of complications, ultimately hindering functional recovery and diminishing long-term QoL in this high-risk population.

For the elderly, particularly those with pre-existing comorbidities or reduced functional capacity, hip fracture leads to lasting consequences, including loss of independence, disruption of daily life, and a substantial decline in QoL [17–20]. However, recovery varies widely among individuals and is influenced by factors such as age, cognitive status, pre-fracture functional level, and postoperative complications [21, 22]. Understanding the patterns of QoL change over time and identifying subgroups with similar recovery profiles are essential for tailoring rehabilitation strategies and improving long-term outcomes [23]. 

The growth mixture modelling (GMM) approach has been employed to evaluate changes in QoL in various clinical settings [24–26]. By modelling longitudinal data, GMM can reveal distinct recovery patterns and between-patient differences that single-time-point or average analyses may overlook. Nevertheless, few studies have used this method to examine longitudinal QoL trajectories in elderly patients after hip fracture [27, 28]. Therefore, in this study, we used GMM to identify and characterize distinct trajectories of QoL following hip fracture surgery, based on assessments at baseline (pre-injury), 6 months, and 1 year postoperatively. Our goal was to uncover subgroups of patients with similar patterns of QoL change and to examine the demographic and clinical factors associated with each trajectory.

## Materials and methods

This longitudinal study was conducted at Wan Fang Hospital, Taipei, Taiwan, between 2019 and 2022, using data from our hip fracture registry. The registry is prospective and single-center, approved by the Taipei Medical University Joint Institutional Review Board (TMU-JIRB No. N201709053), and created explicitly for systematic data collection in the elderly undergoing hip fracture surgery, rather than relying on retrospective chart extraction. Eligible participants were aged 60 years or older, sustained hip fractures (intracapsular or extracapsular fractures) due to low-trauma injuries, and received surgical treatments (osteosynthesis or hip arthroplasty). Exclusion criteria included those receiving hip surgery for non-fracture reasons, fractures caused by high-energy trauma, fractures associated with metastatic disease or primary bone tumors, and cases involving active infections or septic arthritis of the hip. Patients who died within a 1-year follow-up or lacked QoL data at any scheduled assessment were also excluded. The study was conducted in accordance with the Declaration of Helsinki and was approved by the Ethics Committee of our Institute. Written informed consent was obtained from all participants for both study participation and publication. All records were securely stored, de-identified for analysis, and subject to monitoring and audit under our TMU-JIRB oversight to ensure data accuracy, transparency, and Good Clinical Practice compliance.

Data were collected using protocol-specific case report forms derived from standard templates. Variables were predefined before enrollment. Demographic variables included age, sex, and body mass index (BMI). Socioeconomic factors encompassed education level, marital status, and living arrangements. Clinical characteristics included fracture type, whether the fracture was a first or subsequent event, mental status assessed via the Short Portable Mental Status Questionnaire (SPMSQ) [29], QoL was evaluated using the EuroQoL 5-dimension 3-level (EQ-5D-3L) questionnaire and converted into an utility score according to the Taiwanese value set [30, 31], and activities of daily living (ADL) measured by the Barthel Index (BI) [32]. The pre-fracture status was collected through interviews with patients or their caregivers. Comorbidity burden was evaluated via the Charlson Comorbidity Index (CCI). Dual-energy X-ray absorptiometry (DXA) was used to assess musculoskeletal health. Osteoporosis was diagnosed according to the World Health Organization (WHO) criteria, defined as a bone mineral density (BMD) T-score ≤ −2.5 at the lumbar spine [33]. Sarcopenia was diagnosed according to the 2019 consensus of the Asian Working Group for Sarcopenia (AWGS), which requires the presence of low muscle mass in combination with low muscle strength [34]. Appendicular lean mass (ALM) was assessed and adjusted for height squared, with cutoff values of < 7.0 kg/m² for men and < 5.7 kg/m² for women, while muscle strength was assessed by handgrip strength, with cutoffs of < 28 kg for men and < 18 kg for women. Patients having co-occurrence of osteoporosis and sarcopenia were diagnosed with osteosarcopenia. After hip fracture surgery, early mobilization was promoted to reduce complications and support early standing. Patients performed daily strengthening exercises and gradually increased weight-bearing on the non-injured leg, beginning as early as the first postoperative day. Walking training typically started within the first week, once full weight-bearing and balance were tolerated. Rehabilitation also included stair-climbing, fall-prevention strategies, and instruction in using assistive devices. All patients received guidance from physical therapists during hospitalization, with structured rehabilitation continuing for up to three months after discharge. A part of patient enrollment and study methodology has been described in our previous publications [19, 35, 36]. 

Because QoL was the primary outcome, the trajectory analysis included only patients with complete EQ-5D-3L measurements at all three time points; individuals with missing QoL data at any follow-up were excluded. For other variables, missingness was minimal, with only two covariates (BMI and handgrip strength) each having one missing value (< 5%). These were addressed through manual single imputation based on available clinical information to preserve sample size and maintain data consistency.

To examine the trend of QoL from the pre-injury period to 6 months and 1 year after surgery, we applied GMM with a quadratic specification of time (including both linear and squared time terms) to identify distinct QoL trajectories [37]. Since formal a priori sample size calculations for GMM are not available, we evaluated adequacy based on commonly recommended criteria for model stability, including the number of latent classes, class separation, number of repeated measures, and convergence diagnostics. Accordingly, no conventional power calculation was undertaken. Models with one to five latent classes were tested to determine the optimal number of trajectory groups. Model selection was guided by the Bayesian Information Criterion (BIC), Akaike Information Criterion (AIC), class size (minimum 5% of the sample), posterior class-membership probabilities (mean > 0.7), and clinical interpretability [38]. 

After selecting the optimal model, we described the trajectory patterns and estimated each patient’s posterior class-membership probability. Categorical variables were summarized as frequencies and percentages, while continuous variables were presented as means with standard deviations. Comparisons between trajectory groups were made using one-way ANOVA for continuous variables and Chi-square or Fisher’s exact tests for categorical variables. Multivariate multinomial logistic regression was conducted to identify baseline predictors of trajectory group membership, with results reported as odds ratios (ORs) and 95% confidence intervals (CIs).

The trajectory models were fitted using the lcmm package and the hlme() function in R (version 2025.05.1 + 513 “Mariposa Orchid” Release for Windows). Descriptive statistics, group comparisons, and multivariate regression were performed on SPSS Statistics for Windows (version 27.0, IBM Corp.). Statistical significance was defined as *p* < 0.05.

## Results

Out of an initial cohort of 500 patients with hip fractures, 58 were excluded due to death within one year, and 60 were excluded for missing QoL data at the 6-month follow-up, resulting in a final sample of 382 patients. The mean age was 80.23 ± 9.24 years, and the majority were female (*n* = 274, 71.3%), compared to male patients (*n* = 108, 28.3%). Baseline characteristics of the included participants are presented in Table 1.

**Table 1:** Baseline characteristics of the participants

Variables	Mean ± SD or N (%)
Age	80.23 ± 9.24
*Sex*	
Male	108 (28.3)
Female	274 (71.3)
Body mass index	22.61 ± 3.86
Charlson Comorbidity Index	1.18 ± 1.54
*Education*	
Primary school and below	202 (52.9)
Junior high school	52 (13.6)
Senior high school	68 (17.8)
Undergraduate	55 (14.4)
Postgraduate	5 (1.3)
*Marriage*	
Married	253 (66.2)
Widow	105 (27.5)
Unmarried	17 (4.5)
Divorced	7 (1.8)
*Coliving*	
Living with family	325 (85.1)
Living alone	35 (9.2)
Nursing care center	22 (5.8)
*Fracture types*	
Intracapsular fracture	243 (63.6)
Extracapsular fracture	139 (36.4)
*Hip fracture surgery*	
Hip arthroplasty	171 (44.8)
Osteosynthesis	211 (55.2)
*Second hip fracture*	
Yes	26 (6.8)
No	356 (93.2)
*Osteosarcopenia*	
Yes	155 (40.6)
No	227 (59.4)
*Quality of life*	
EQ-5D-3L utility score	0.90 ± 0.16
*Mental status*	
SPMSQ	2.40 ± 3.20
*Activities of daily living*	
Barthel index	89.57 ± 18.04

BMI: Body mass index; CCI: Charlson comorbidity index; EQ-5D-3L: EuroQoL 5-dimension 3-level; SPMSQ: Short portable mental status questionnaire; ADL: Activity of daily living; BI: Barthel Index; SD: Standard deviation

**Fig. 1:**
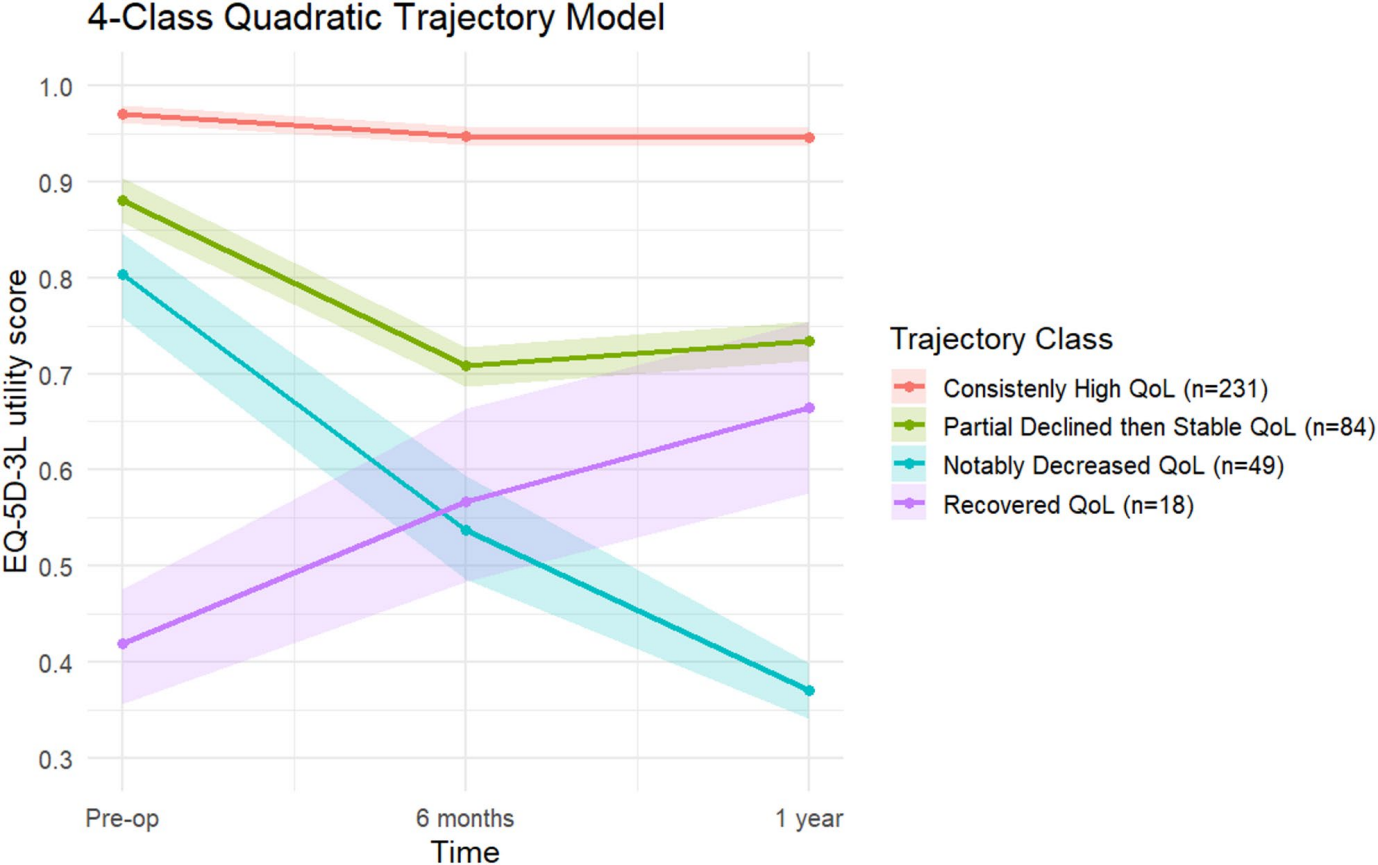
Trajectories of quality of life over time for four classes. Lines represent the mean EQ-5D-3L utility score at each time point (preoperative, 6 months, and 1 year), with shaded ribbons indicating bootstrapped 95% confidence intervals. * Abbreviations* QoL: Quality of life; EQ-5D-3L: EuroQoL 5-dimension 3-level

In Table 2, based on model fit indices and classification quality, the 4-class quadratic model was selected as the optimal solution. It demonstrated the lowest BIC (−1170.539) and AIC (−1237.611), with an acceptable entropy value of 0.86 and robust average posterior probabilities for all classes (> 0.80). Therefore, the 4-class model provided the best balance between fit and interpretability. The four identified trajectories were as follows (Fig. 1). The *consistently high QoL* group (n = 231, 60.47%) showed very high pre-operative QoL (~ 0.97), only a slight decline at 6 months, and stable recovery by 1 year. The *partially declined then stable QoL* group (n = 84, 21.99%) began with relatively high QoL (~ 0.88), showed a moderate decline at 6 months, and then regained some functioning by 1 year, reflecting a temporary postoperative setback. The *notably decreased QoL* group (n = 49, 12.83%) experienced a sharp drop from baseline (~ 0.80) to 6 months (~ 0.46), followed by further deterioration at 1 year (~ 0.38), representing the poorest recovery. Finally, the *recovered QoL* group (n = 18, 4.71%) began with the lowest baseline QoL (~ 0.42) but showed continuous improvement, reaching ~ 0.66 by 1 year, indicating substantial postoperative recovery despite a poor initial status.

**Table 2:** Comparison of model fit statistics and classification quality for 2- to 5-class trajectory models

Model	BIC	AIC	LogLik	Entropy	Average posterior probabilities
2-class	−1065.882	−1101.391	559.696	0.878	Class1: 0.979
					Class2: 0.905
3-class	−1153.544	−1204.834	615.417	0.926	Class 1: 0.924
					Class 2: 0.980
					Class 3: 0.926
4-class	−1170.539	−1237.611	635.806	0.860	Class 1: 0.934
					Class 2: 0.947
					Class 3: 0.931
					Class 4: 0.862
5-class	−1146.795	−1229.649	635.824	0.651	Class 1: 0.951
					Class 2: 0.876
					Class 3: NaN
					Class 4: 0.504
					Class 5: 0.936

BIC: Bayesian information criterion; AIC: Akaike information criterion; LogLik: Log-likelihood

NaN indicates undefined or unstable classification estimates for that class

Table 3 demonstrates baseline characteristic comparisons between the four groups. Age differed significantly between classes (*p* < 0.001). Patients in the *consistently high QoL* group were the youngest on average (78.1 years old), whereas those in the other three groups were older. The CCI also differed significantly (*p* = 0.009). The *consistently high QoL* group had the lowest comorbidity burden, while all other groups showed higher comorbidity levels. Coliving status was associated with trajectory membership (*p* = 0.038). Patients with *notably decreased QoL* and those with *recovered QoL* were more likely to reside in nursing institutions than the other groups, whereas living with family was most common in the *consistently high QoL* group. The presence of osteosarcopenia showed a strong association with QoL class (*p* < 0.001). The prevalence was lowest in the *consistently high QoL* group (32.5%) but markedly higher in the *notably decreased QoL* (63.3%) and *partially declined then stable QoL* (48.8%) groups. SPMSQ scores differed significantly (*p* < 0.001). Cognitive impairment was mild in the *consistently high QoL* (mean of 1.24) group but more pronounced in the *notably decreased*,* partially declined then stable QoL*, and especially the *recovered QoL* group.

**Table 3:** Baseline characteristic comparisons between the four trajectories

Variables	Consistently high QoL n = 231	Notably decreased QoL n = 49	Partially declined then stable QoL n = 84	Recovered QoL n = 18	*P*-value
Age	78.08 ± 9.22	83.47 ± 8.26	83.33 ± 8.19	84.44 ± 9.19	< 0.001*
*Sex*					
Male	69 (29.9)	16 (32.7)	19 (22.6)	4 (22.2)	0.503
Female	162 (70.1)	33 (67.3)	65 (77.4)	14 (77.8)	
Body mass index	22.87 ± 3.91	21.53 ± 3.63	22.34 ± 3.39	23.45 ± 5.38	0.105
Charlson comorbidity index	0.97 ± 1.38	1.53 ± 1.82	1.45 ± 1.68	1.67 ± 1.68	0.009*
*Education*					
Primary school and below	118 (51.1)	24 (49.0)	50 (59.5)	10 (55.6)	0.681
Junior high school	27 (11.7)	10 (20.4)	12 (14.3)	3 (16.7)	
Senior high school	47 (20.3)	10 (20.4)	9 (10.7)	2 (11.1)	
Undergraduate	35 (15.2)	5 (10.2)	12 (14.3)	3 (16.7)	
Postgraduate	4 (1.7)	0 (0)	1 (1.2)	0 (0)	
*Marriage*					
Married	159 (68.8)	29 (59.2)	55 (65.5)	10 (55.6)	0.139
Widow	52 (22.5)	17 (34.7)	28 (33.3)	8 (44.4)	
Unmarried	14 (6.1)	2 (4.1)	1 (1.2)	0 (0)	
Divorced	6 (2.6)	1 (2.0)	0 (0)	0 (0)	
*Coliving*					
Living with family	200 (86.6)	39 (79.6)	73 (86.9)	13 (72.2)	0.038*
Living alone	23 (10.0)	4 (8.2)	7 (8.3)	1 (5.6)	
Nursing care institutions	8 (3.5)	6 (12.2)	4 (4.8)	4 (22.2)	
*Fracture patterns*					
Intracapsular fracture	154 (66.7)	27 (55.1)	52 (61.9)	10 (55.6)	0.391
Extracapsular fracture	77 (33.3)	22 (44.9)	32 (38.1)	8 (44.4)	
*Hip fracture surgery*					
Hip arthroplasty	108 (46.8)	20 (40.8)	36 (42.9)	7 (38.9)	0.796
Osteosynthesis	123 (53.2)	29 (59.2)	48 (57.1)	11 (61.1)	
*Second hip fracture*					
Yes	17 (7.4)	1 (2.0)	5 (6.0)	3 (16.7)	0.189
No	214 (92.6)	48 (98.0)	79 (94.0)	15 (83.3)	
*Osteosarcopenia*					
Yes	75 (32.5)	31 (63.3)	41 (48.8)	8 (44.4)	< 0.001*
No	156 (67.5)	18 (36.7)	43 (51.2)	10 (55.6)	
*Quality of life*					
EQ-5D-3L utility score	0.97 ± 0.07	0.80 ± 0.16	0.88 ± 0.11	0.42 ± 0.13	< 0.001*
*Mental status*					
SPMSQ	1.24 ± 2.32	4.18 ± 3.80	3.69 ± 3.31	6.33 ± 3.16	< 0.001*
*Activities of daily living*					
Barthel index	97.53 ± 6.31	74.90 ± 22.51	84.35 ± 17.56	51.67 ± 26.12	< 0.001*

* Statistically significant

QoL: Quality of life; EQ-5D-3L: EuroQoL 5-dimension 3-level; SPMSQ: Short portable mental status questionnaire

Multivariate multinomial logistic regression was conducted to identify factors associated with membership in each QoL trajectory, using the *consistently high QoL* class as the reference group (Table 4). Based on the preliminary group-comparison analysis, seven variables that showed statistically significant differences across trajectories (age, CCI, coliving status, osteosarcopenia, baseline EQ-5D-3L score, SPMSQ, and Barthel Index) were included in the regression model. The analysis showed that patients in the *notably decreased QoL* trajectory were more likely to have osteosarcopenia (OR = 0.317; 95% CI 0.139–0.722), lower baseline QoL (OR = 0.004; 95% CI 5.7E-5–0.286), and poorer functional independence (BI OR = 0.915; 95% CI 0.880–0.952). Membership in the *partially declined then stable QoL* group was associated with older age (OR = 1.037; 95% CI 1.000–1.076), worse cognitive function (SPMSQ OR = 1.138; 95% CI 1.023–1.265), lower preoperative QoL (OR = 0.120; 95% CI 0.002–6.012), and reduced functional independence (Barthel Index OR = 0.928; 95% CI 0.894–0.963). No covariates were statistically significant in predicting membership in the *recovered QoL* trajectory, although point estimates suggested that these patients tended to have lower baseline QoL and higher comorbidity burden before surgery. Overall, lower preoperative QoL, osteosarcopenia, impaired cognition, and poorer functional status were the strongest factors differentiating the poorer trajectories from the *consistently high QoL* group.

**Table 4:** Multivariate multinomial logistic regression predicting the trajectory of QoL among the elderly undergoing hip fracture surgery

Variables	OR (95% CI)			
	Consistently high QoL	Notably decreased QoL	Partially declined then stable QoL	Recovered QoL
Age	Ref	1.044 (0.995–1.095)	**1.037 (1.000-1.076)**	1.481 (0.936–2.343)
Charlson comorbidity index	Ref	1.249 (0.979–1.593)	1.179 (0.982–1.415)	7.933 (0.969–64.927)
*Coliving*				
Living with family	Ref	0.521 (0.122–2.224)	1.368 (0.331–5.653)	12.211 (0.211-705.471)
Living alone		0.801 (0.112–5.259)	1.437 (0.265–7.790)	11.749 (1.966E-5 to 70220145.539)
Nursing care institutions		Ref	Ref	Ref
*Osteosarcopenia*				
No	Ref	**0.317 (0.139–0.722)**	0.647 (0.354–1.185)	0.037 (0.000-4.950)
Yes		Ref	Ref	Ref
*Quality of life*				
EQ-5D-3L utility score	Ref	**0.004 (5.736E-5 to 0.286)**	0.120 (0.002–6.012)	**6.121E-27 (1.606E-48 to 2.334E-5)**
*Mental status*				
SPMSQ	Ref	1.079 (0.944–1.233)	**1.138 (1.023–1.265)**	0.770 (0.407–1.457)
*Activities of daily living*				
Barthel index	Ref	**0.915 (0.880–0.952)**	**0.928 (0.894–0.963)**	0.926 (0.840–1.021)

* Statistically significant

Bold font indicates statistical significance

EQ-5D-L: EuroQoL 5-dimension 3-level; SPMSQ: Short portable mental status questionnaire

## Discussions

In this cohort of elderly undergoing hip fracture surgery, we identified four distinct postoperative QoL trajectories using quadratic growth mixture modelling. Most patients followed a *consistently high QoL* pattern with minimal decline and strong recovery, whereas smaller subgroups demonstrated *partially declined then stable QoL*,* notably decreased QoL*, or *recovered QoL*. Multivariate multinomial regression showed that osteosarcopenia, lower pre-fracture EQ-5D-3L, and poorer activity of daily living predicted the *notably decreased QoL* trajectory, while older age and worse cognition predicted the *partially declined then stable* trajectory. No baseline covariates significantly predicted the recovered trajectory.

The trajectories in our study illustrate how changes in QoL over time can shape patient outcomes. The *consistently high QoL* trajectory, comprising the majority of patients, represents a resilient group that experienced only minimal QoL loss and maintained high QoL, consistent with a previous study showing that minimal short-term QoL loss predicts better long-term QoL after hip fracture surgery [36]. The *partially declined then stable* trajectory captures patients who experience a moderate drop in QoL by 6 months but subsequently stabilize, suggesting a “delayed but steady” recovery pattern commonly observed in frail patients undergoing prolonged rehabilitation [39–41]. The *recovered QoL* trajectory, although small, highlights an important subgroup with poor preoperative QoL who showed substantial and continuous improvement, reinforcing the idea that even highly vulnerable patients may achieve meaningful gains from surgeries after their hip fractures. The identification of a *notably decreased QoL* trajectory in our study offers a new perspective compared to previous literature, which has primarily described stable QoL patterns categorized as poor, moderate, or good [27, 28]. In those studies, these trajectories typically remained relatively flat across follow-up periods, suggesting that patients’ recovery outcomes were stable. Together, these four trajectories emphasize that postoperative QoL is not uniform, and early deviations, especially excessive decline within the first 6 months, may signal long-term vulnerability. Identifying these patterns early offers an opportunity to tailor postoperative care, enhance rehabilitation intensity, and prioritize interventions for those most at risk.

The multivariate multinomial logistic regression analysis revealed several independent predictors that consistently differentiated the four QoL trajectories, and each predictor provides meaningful insight into the underlying recovery mechanisms following hip fracture surgery. Age was a significant predictor only for the *partially declined then stable* trajectory, but not for the *notably decreased* or *recovered* groups, suggesting that age influences QoL recovery in a selective, non-linear way [42]. Age did not even predict the poorest trajectory, implying that other factors may drive the ongoing QoL deterioration in this group.

Patients with impaired ADL were more likely to follow poorer QoL trajectories. ADL plays an essential role in determining functional recovery and long-term outcomes in patients following hip fracture [43–45]. Hip fracture patients also reported that they would have better QoL if they had more baseline ADL [10]. Patients who maintain or regain the ability to perform self-care, mobility, and domestic tasks independently are less reliant on caregivers, experience greater autonomy, and retain a sense of control over their lives. This independence facilitates social participation and reduces the psychological burden of dependency, which can mitigate depressive symptoms and enhance life satisfaction [46]. However, as ADL reflects long-standing health conditions and is not easily modifiable in the short term, it may serve as a better tool for risk stratification and prognosis, helping to identify patients at greater risk for QoL decline after hip fracture surgery.

Cognitive status demonstrated a selective influence on trajectory membership. Our result indicated that patients with more severe cognitive impairment were more likely to follow the *partially declined then stable* pattern. This aligns with prior studies showing that patients with mild cognitive deficits often experience delayed functional improvement but can still stabilize as rehabilitation progresses [47, 48]. Interestingly, SPMSQ was not a significant predictor of *notably decreased* or *recovered* trajectories, suggesting that baseline cognition alone does not fully explain the most extreme QoL outcomes.

Osteosarcopenia was identified as a significant and potentially modifiable factor associated with poorer QoL after hip fracture surgery, consistent with prior studies highlighting its detrimental impact on health and well-being in older adults [49–54]. The loss of bone and muscle mass increases fracture susceptibility, initiating a cascade of physical, psychological, and social consequences [55, 56]. Beyond impaired mobility, osteosarcopenia frequently causes chronic or recurrent pain, which can restrict daily activities, diminish self-efficacy, and exacerbate both physical and mental health burdens [57, 58]. In addition, the heightened fear of falling common among individuals with osteosarcopenia often leads to activity restriction and social withdrawal, further reducing physical conditioning and emotional well-being [59–61]. These factors can substantially hinder the likelihood of regaining pre-fracture functional levels. Notably, previous studies have shown that management of osteosarcopenia not only improves bone health and reduces refracture risk but also alleviates psychological distress and enhances QoL in osteoporotic patients [61–66]. Therefore, routine assessment of musculoskeletal health should be integrated into the care pathway for hip fracture patients, with initiation of treatment of osteosarcopenia during hospitalization or early in the postoperative period, alongside the implementation of a structured secondary fracture prevention program [65, 67]. 

Our findings highlight a nuanced role of baseline QoL in predicting postoperative trajectories, although it emerged as one of the strongest predictors of trajectory membership. Compared with the *consistently high QoL* group, patients with *notably decreased QoL*, who had a modest reduction in QoL, had dramatically lower odds of belonging to the high-QoL trajectory, indicating that poor pre-fracture health status strongly predisposes patients to sustained deterioration after hip fracture. Paradoxically, patients with the lowest baseline QoL were more likely to follow the *recovered QoL* trajectory. Hip fracture surgery may serve as a reset, enabling gains that exceed pre-injury status [68]. These contrasting patterns indicate that while mildly impaired baseline QoL signals risk for poor recovery, severely reduced baseline QoL may identify patients who stand to benefit the most from surgical intervention and structured rehabilitation.

This study has some limitations that warrant consideration. First, it was conducted at a single medical center, which may limit the generalizability of the findings to other healthcare settings and populations. Post-fracture QoL trajectories are likely shaped not only by clinical factors but also by cultural norms and healthcare system characteristics, such as the availability and intensity of rehabilitation, family involvement in caregiving, and expectations regarding functional independence in the elderly. Second, pre-fracture status was obtained retrospectively from patients or caregivers during hospital admission, which may introduce recall bias, particularly among individuals with cognitive impairment. Third, while we utilized GMM to account for heterogeneity in QoL trajectories, the model is sensitive to class specification and starting values. Although we followed established criteria for model selection and demonstrated high classification quality, alternative trajectory structures may exist. Fourth, because only three postoperative time points were available (preoperative, 6 months, and 1 year), the trajectory models should be interpreted as exploratory. While the quadratic specification allowed us to capture curvature in recovery patterns, additional follow-up assessments would improve the model’s ability to delineate more complex or fluctuating trajectories. Future studies with more frequent longitudinal measurements are needed to validate and refine these trajectory classes. Lastly, we collected data 1 year after surgery and evaluated QoL at 3 time points, which may not fully capture the recovery process or potential fluctuations over time.

Nonetheless, the use of dynamic trajectory modelling in this study provides valuable insight into individual differences in recovery patterns and supports the development of more personalized care strategies for the elderly following hip fracture. Given the limited number of studies investigating QoL trajectories in this population, we hope these findings contribute meaningfully to the growing body of evidence in geriatric orthopedic care.

## Conclusion

Four QoL trajectories within one year following hip fracture surgery in elderly patients were explored. Age, comorbidity burden, musculoskeletal health, cognitive status, baseline QoL, and functional independence were significantly associated with trajectory membership. Routine assessment of these factors can help guide personalized management to help maintain QoL and prevent further decline.

## Data Availability

No datasets were generated or analysed during the current study.

## Abbreviations

QoL: Quality of life
GMM: Growth mixture modelling
BMI: Body mass index
SPMSQ: Short portable mental status questionnaire
EQ-5D-3L: EuroQoL 5-dimension 3-level
ADL: Activities of daily living
BI: Barthel index
CCI: Charlson comorbidity index
DXA: Dual-energy X-ray absorptiometry
WHO: World Health Organization
AWGS: The Asian Working Group for Sarcopenia
ALM: Appendicular lean mass
BIC: Bayesian Information Criterion
AIC: Akaike Information Criterion
Loglik: Log-likelihood
OR: Odds ratio
CI: Confidence interval
